# Cell-type-specific actions of carnosic acid against *Porphyromonas gingivalis*-induced proliferation in liver cancer cells

**DOI:** 10.1080/20002297.2026.2684127

**Published:** 2026-06-13

**Authors:** Ting Wang, Toshimi Chiba, Taichi Ishikawa, Hidekatsu Kuroda, Keisuke Kakisaka

**Affiliations:** a Division of Internal Medicine, Department of Oral Medicine, Iwate Medical University, Iwate, Japan; b Division of Molecular Microbiology, Department of Microbiology, Iwate Medical University, Morioka, Japan; c Division of Hepatology, Department of Internal Medicine, Iwate Medical University School of Medicine, Iwate, Japan

**Keywords:** *Porphyromonas gingivalis*, lipopolysaccharide, oncogenic effects, liver cancer, carnosic acid, Nrf2

## Abstract

**Background and aim:**

The periodontal pathogen *Porphyromonas gingivalis* (Pg) has been linked to systemic diseases, including cancer.

**Objective:**

To investigate the oncogenic effects of Pg and its lipopolysaccharide (LPS) on liver cancer cells and evaluate the anti‑cancer potential of carnosic acid (CA).

**Design:**

HepG2, HuH7 and pancreatic cancer cells were treated with Pg, Pg‑LPS or CA. Proliferation was assessed by viability assay. Key phosphorylated signaling molecules and their total proteins (TLR4, ERK, Akt, NF‑κB, Nrf2) were examined by western blot and immunofluorescence.

**Results:**

Pg promoted proliferation in HepG2 and pancreatic cancer cells in an MOI‑dependent manner and upregulated TLR4, p‑ERK, p‑Akt and p‑NF‑κB. Pg‑LPS enhanced proliferation, but CA did not inhibit this effect. In HuH7 cells, CA inhibited proliferation, associated with Nrf2 activation; in HepG2 cells, CA showed no inhibition or Nrf2 activation.

**Conclusions:**

Pg promotes oncogenic processes. CA modulates proliferation in a cell‑type‑ and concentration‑dependent manner, likely through Nrf2 activation.

## Introduction

Liver cancer remains a major global health concern and is among the leading causes of cancer-related mortality [1]. Hepatocellular carcinoma (HCC), the most common form of primary liver cancer, often arises from chronic liver conditions, including hepatitis B and C infections, alcohol-induced liver damage and non-alcoholic fatty liver disease (NAFLD) [2]. NAFLD is associated with metabolic disorders such as obesity and diabetes, and its progression to non-alcoholic steatohepatitis (NASH) significantly increases the risk of cirrhosis and HCC [3].

*Porphyromonas gingivalis* (Pg) is a Gram-negative anaerobic bacterium primarily associated with periodontitis. However, recent studies indicate that Pg can affect systemic health, contributing to liver diseases and cancer [4,5]. Pg has been detected in various organs, suggesting it can translocate and impact other tissues, potentially promoting carcinogenesis through chronic inflammation and immune modulation [6]. Pg's virulence factors, particularly lipopolysaccharide (LPS), activate Toll-like receptors (TLRs) such as TLR4, leading to pro-inflammatory and oncogenic signaling [7,8]. LPS activation of TLR4 promotes the phosphorylation of ERK and Akt, pathways crucial for proliferation, survival and migration, which are key processes in cancer development [9,10].

The role of Pg in liver diseases, especially NAFLD, is of significant interest. Chronic inflammation is central to NAFLD progression from benign fatty liver to HCC. Pg and other periodontal pathogens have been reported to exacerbate liver inflammation, accelerating NAFLD's transition to HCC [11]. Pg-induced inflammation can drive cytokine production, including interleukin-6 (IL-6) and tumor necrosis factor-alpha (TNF-*α*), which contributes to an inflammatory hepatic environment that fosters cancer development [12]. Furthermore, Pg translocation from the oral cavity to the liver, via the bloodstream or lymphatic pathways, offers a plausible mechanism for its direct impact on liver pathology [6,13].

There are limited effective therapies to counteract Pg's pathogenic effects on liver diseases. Completely eliminating Pg is challenging, and antibiotic use often has severe side effects. Thus, there is interest in finding therapeutic agents that mitigate these effects. Natural compounds with anti-inflammatory and antioxidant properties, like carnosic acid (CA), are potential adjuncts in cancer therapy. CA, a phenolic diterpene found in rosemary and sage, has shown antioxidative and anti-inflammatory activities through modulation of pathways like Nrf2 [14–16]. In liver cancer, CA has demonstrated benefits such as ameliorating NAFLD-related liver damage and preventing NASH progression [16–18]. Notably, research suggests CA can exert anti-proliferative and pro-apoptotic effects in various cancer models, highlighting its potential as a modulator of tumor growth [19,20]. By targeting key inflammatory and oxidative stress pathways, CA shows promise in modulating the liver cancer microenvironment. Based on the above studies, it is conceivable and worth validating that CA exerts an inhibitory effect on Pg-induced carcinogenesis.

This study first confirmed the proliferative effects of *Pg* or its LPS on HepG2 cells (with pancreatic cells as a positive control). We then evaluated whether CA could counteract these effects. However, the initial results in HepG2 cells did not meet our expectations. To determine whether CA's action is bacteria-driven or cell-type-dependent, we extended our analysis to HuH7 cells, focusing on the Nrf2 pathway.

## Materials and methods

### Cell cultures

HepG2 hepatocellular carcinoma cells (ATCC, Manassas, VA, USA), along with control cells such as human pancreatic carcinoma cells (RCB 1973: PK-45H, RIKEN BRC, Japan) and HuH7 liver cancer cells (ATCC, Manassas, VA, USA), were used in this study. HepG2 and HuH7 cells were cultured in Dulbecco's Modified Eagle Medium (DMEM; Gibco, Thermo Fisher Scientific, Waltham, MA, USA), while human pancreatic carcinoma cells were cultured in RPMI 1640 medium (Gibco, Thermo Fisher Scientific, Waltham, MA, USA). All media were supplemented with 10% fetal bovine serum (FBS; Sigma-Aldrich, St. Louis, MO, USA) and antibiotics. The cells were maintained at 37 °C in a humidified atmosphere containing 5% CO₂.

### Pg and Pg-derived LPS treatment

Pg was grown anaerobically, and a range of multiplicities of infection (MOIs; 0.01–10) was used to evaluate dose-dependent effects of Pg, covering conditions from low bacterial burden to biologically active levels 0 to 10 were applied to the cancer cells for specified durations. Pg-derived LPS was purchased from Merck (Darmstadt, Germany). In a preliminary test, cells were treated with LPS at concentrations ranging from 0.01 to 10 µg/mL to evaluate its effects on cell proliferation; 1 µg/mL was selected for the study.

### CA treatment

Carnosic acid (CA) was obtained from Sigma-Aldrich (St. Louis, MO, USA), dissolved in dimethyl sulfoxide (DMSO) and then diluted to final concentrations ranging from 0.1 to 10 µM.

### Cell proliferation assay

A live cell counting assay was performed using Cell Count Reagent SF (Nacalai Tesque Inc., Kyoto, Japan). Cells were seeded into 96-well plates at a density of 5,000 cells per well and treated with varying doses of Pg, Pg-derived LPS, CA, or their combinations. After 24 hours of incubation, the SF reagent was added to each well, and the cells were incubated for an additional hour. Absorbance was then measured at 450 nm using a microplate photometer (IMMUNO-MINI NJ-2300, Inter Med Co., Tokyo, Japan).

### Western blot analysis

Cells were lysed using RIPA buffer, and protein concentrations were quantified using the BCA Protein Assay Kit (Thermo Fisher, Waltham, MA, USA). Equal amounts of protein were separated by SDS-PAGE and transferred to nitrocellulose membranes. The membranes were probed with primary antibodies against TLR4, Nrf2 (Abcam, Cambridge, UK), ERK, Akt, NF-κB, *p*-ERK, *p*-Akt and *p*-NF-κB (Cell Signaling Technology, CST, Danvers, MA, USA). Bands were visualized using enhanced chemiluminescence (ECL) and quantified with ImageJ software.

### Immunofluorescence

For detecting Nrf2 translocation, cells were fixed with 4% paraformaldehyde, permeabilized with Triton X-100 and incubated with an anti-Nrf2 antibody followed by an Alexa Fluor-conjugated secondary antibody. Nuclei were counterstained with DAPI (CST, Danvers, MA, USA). Nrf2 nuclear translocation was quantified as Cells with nuclear Nrf2 (%) using ImageJ (≥50 cells/group). Data were analyzed by two-tailed Student's t-test and expressed as mean ± SD. Additionally, to detect live and dead cells, cells were stained with the Live/Dead Cell Viability Kit (Thermo Fisher Scientific, Waltham, MA, USA). This kit detects compromised membrane integrity via propidium iodide (PI) uptake and therefore directly measures cell death.

Images were captured using a fluorescence microscope.

### Statistical analysis

Three independent experiments were performed, each with technical replicates (*n* = 3). All data are expressed as mean ± standard deviation (SD). One-way analysis of variance (ANOVA) was performed, followed by Tukey's post hoc test for all pairwise comparisons or Dunnett's post hoc test for comparisons against a single control group. A *p*-value < 0.05 was considered statistically significant. All statistical analyzes were conducted using SPSS Statistics version 26.0 (IBM Corp., Armonk, NY, USA).

## Results

### Pg promotes the proliferation of pancreatic cancer cells and HepG2 cells

**Figure 1.**
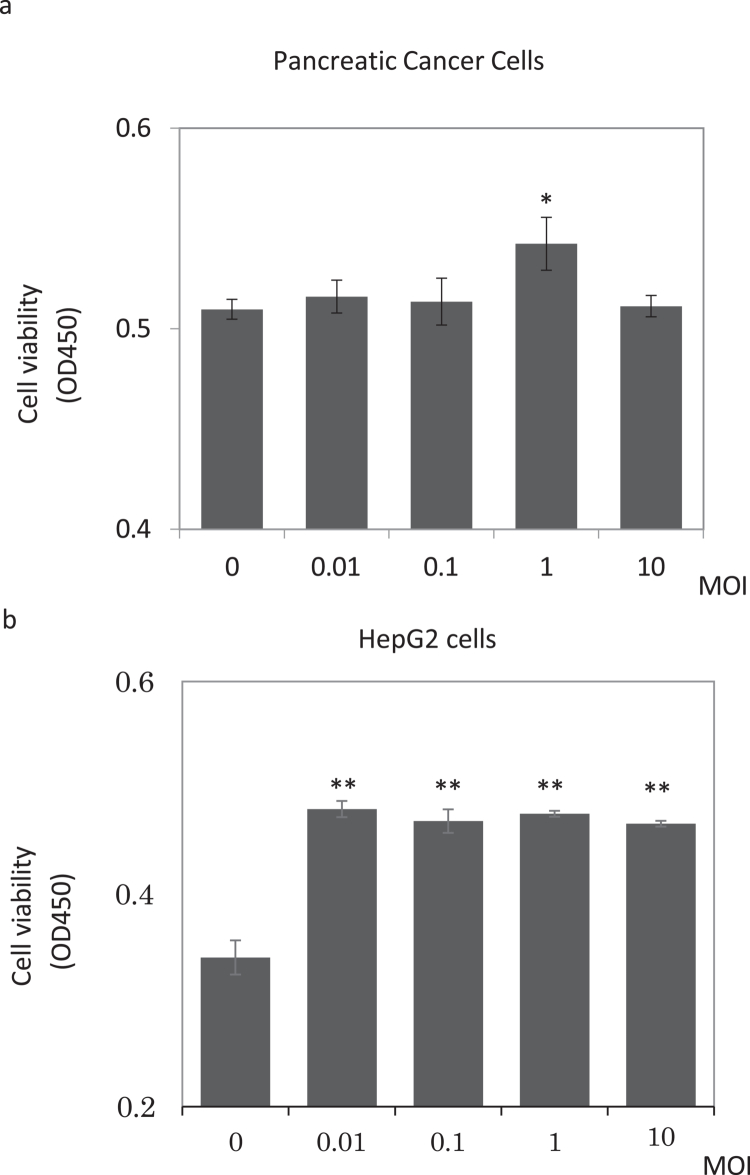
Effects of Pg on cell proliferation. The proliferation of pancreatic cancer cells (a) and HepG2 cells (b) treated with varying multiplicities of infection (MOI) of Pg was measured using cell viability assays as described in the Methods. (c) Data are expressed as the mean ± SD (*n* = 3). *, *p* < 0.05; **, *p* < 0.01 vs. control (MOI 0). One-way ANOVA with Dunnett's post hoc test was used.

We first examined the effects of Pg on the proliferation of HepG2 cells and pancreatic cancer cells, the latter serving as a positive control due to their shared origin as gastrointestinal tract-derived cancers. Cells were treated with Pg at multiplicities of infection (MOI) ranging from 0 to 10. Pg significantly increased the proliferation of pancreatic cancer cells at an MOI of 1 (Figure 1a). In HepG2 cells, Pg enhanced proliferation at an MOI as low as 0.01, and this effect was observed across all MOIs tested (Figure 1b).

### Effects of Pg on protein levels of signaling molecules related to proliferation

**Figure 2.**
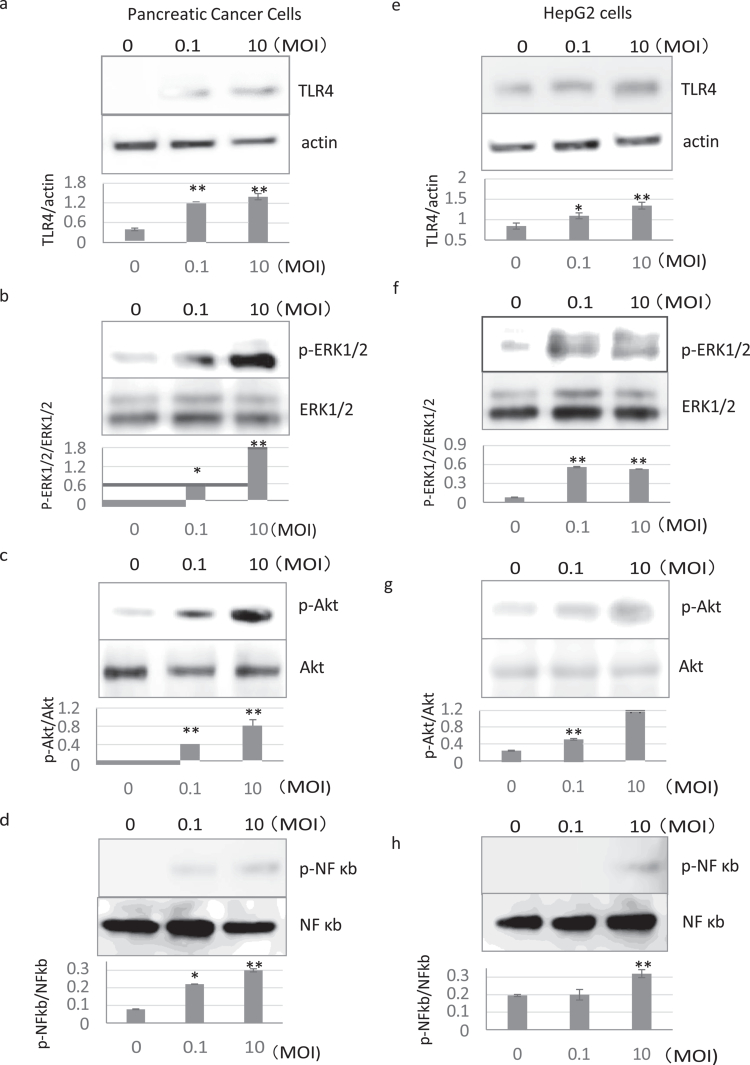
Activation of TLR, ERK, Akt and NF-κB signaling pathways in response to Pg treatment. Western blot analysis of the protein levels of TLR4, *p*-ERK, *p*-Akt and *p*-NF-κB in pancreatic cancer cells and HepG2 cells treated with Pg is shown. The total protein level of the respective protein was blotted as the internal control. The ratios of arbitrary values of protein levels of TLR4 (a, e), *p*-ERK (b, f), *p*-Akt (c, g) and *p*-NF-κB (d, h) against the internal control are shown right under the respective western blotting bands. Data are expressed as the mean ± SD (*n* = 3). Tukey's post hoc test was used.

To explore the key signaling molecules involved in Pg-induced proliferation, we examined the intracellular protein levels of TLR4, *p*-ERK, *p*-Akt and *p*-NF-κB. Compared to controls, treatment with Pg at MOI 0.1 significantly increased protein levels of TLR4 (Figure 2a and e), *p*-ERK (Figure 2b and f) and *p*-Akt (Figure 2c and g) in both pancreatic cancer and HepG2 cells, as well as *p*-NF-κB in pancreatic cancer cells (Figure 2d). At a higher MOI of 10, further enhanced the expression of all these molecules, including *p*-NF-κB, in both pancreatic cancer and HepG2 cells (Figure 2a–h).

### Pg-derived LPS induces proliferation, but CA shows no inhibition in HepG2 cells

**Figure 3.**
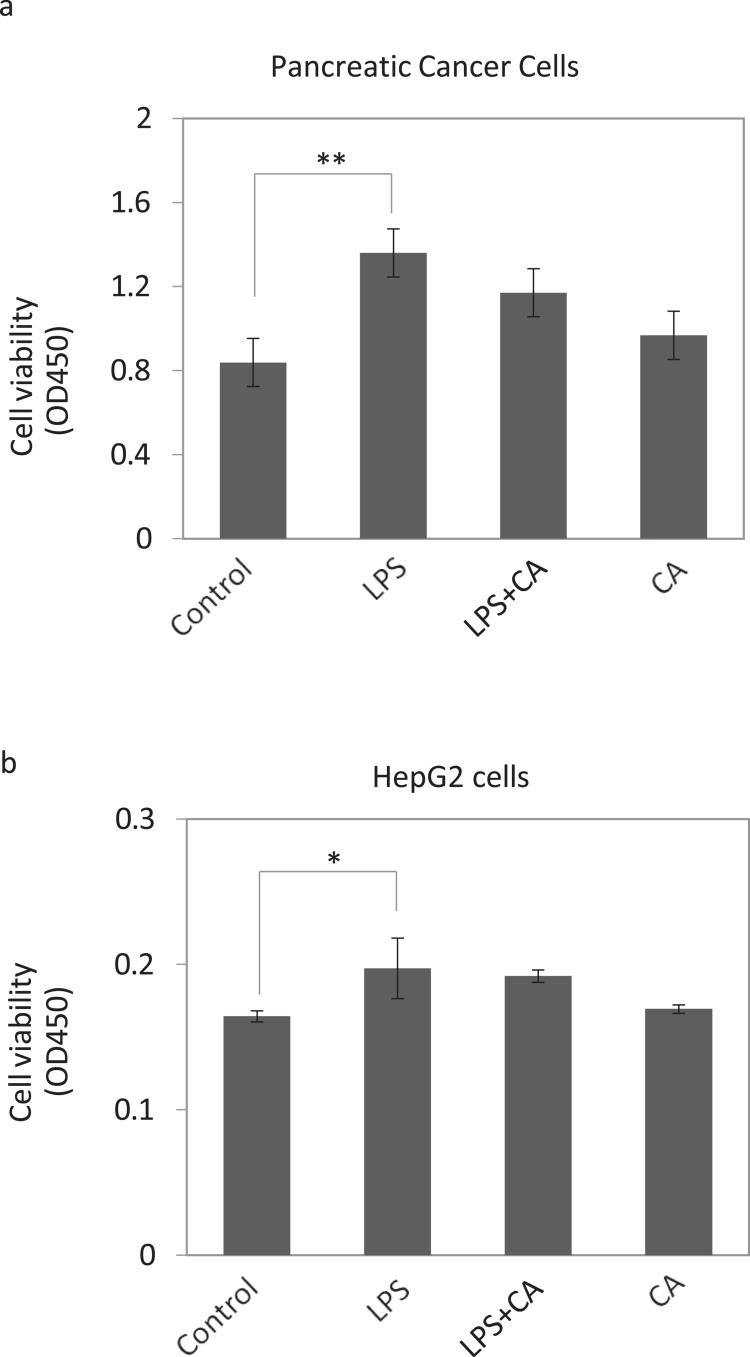
Effects of LPS derived from Pg and CA on cell proliferation. Proliferation of pancreatic cancer cells (a) and HepG2 cells (b) treated with 1 µg/mL Pg-derived LPS alone and in combination with 10 µM CA was detected as described in the Methods. Data are expressed as the mean ± SD (*n* = 3). *, *p* <  0.05 vs. control. **, *p* < 0.01 vs. control. Tukey's post hoc test was used.

We next evaluated whether CA could counteract the proliferative effects of Pg. Given that LPS is the key endotoxin of Pg and avoids live-bacteria interference, we chose Pg-derived LPS instead of live Pg. LPS treatment alone significantly increased proliferation in both pancreatic cancer and HepG2 cells. However, co-treatment with CA did not significantly reduce this LPS-induced proliferation in either cell line (Figure 3a and b).

### Differential effects of CA on HepG2 and HuH7 cells: cell-type dependency

**Figure 4.**
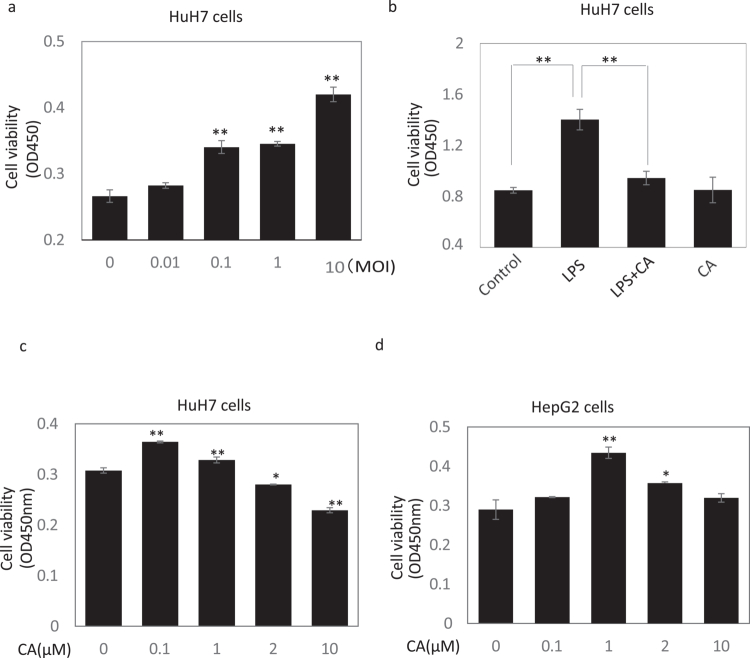
Differential effects of CA on HepG2 and HuH7 cell proliferation: cell type dependency. (a) Proliferation of HuH7 cells stimulated with Pg at the indicated MOI and (b) proliferation of HuH7 cells under four conditions (control, LPS, LPS + CA and CA alone). (c) Proliferation of HuH7 cells and (d) HepG2 cells treated with CA at concentrations ranging from 0.1  µM to 10  µM. All proliferation assays were performed as described in the Methods. Data are expressed as mean ± SD (*n* = 3). *, *p* < 0.05; **, *p* < 0.01 vs. control. One-way ANOVA with Dunnett's post hoc test was used for (a), (c) and (d). Tukey's post hoc test was used for (b).

To test whether the lack of CA effect in HepG2 cells was due to bacterial suppression of CA's action or reflected cell-type specificity, we extended our analysis to HuH7 liver cancer cells. Although both HepG2 and HuH7 cells exhibited increased proliferation upon Pg or LPS stimulation (Figures 1b, 3b, 4a and b), CA produced opposite effects on the two cell lines irrespective of the presence of LPS (Figure 4c and d): it had no inhibitory effect on HepG2 cells, yet it effectively inhibited proliferation in HuH7 cells (CA ≥ 2μM). This observation suggests that CA's action is independent of bacterial effects and led us to investigate the underlying mechanisms, with a focus on the Nrf2 pathway.

### CA induces Nrf2 translocation in HuH7 but not HepG2 cells

**Figure 5.**
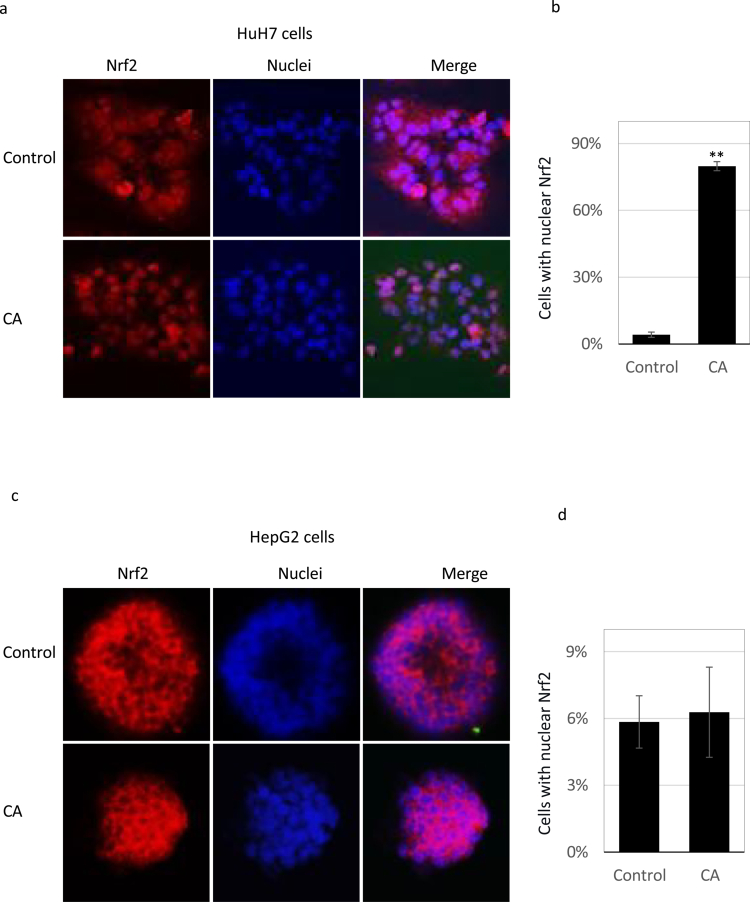
Nrf2 nuclear translocation in response to CA treatment. Immunofluorescence staining of HuH7 cells (a) and HepG2 cells (c) treated with 10 µM CA, showing Nrf2 (red) and nuclei (blue). Overlap indicates Nrf2 translocation to the nucleus. Quantification of cells with nuclear Nrf2 (%) is shown in (b) and (d). Data are mean ± SD (*n* = 3). **p < 0.01 vs. control. Unpaired two-tailed Student's t-test was used.

To investigate the mechanism underlying the differential effects of CA on HepG2 and HuH7 cells, we examined Nrf2 translocation, a key event in the activation of CA-induced signaling pathways. Immunofluorescence staining revealed that CA treatment led to Nrf2 nuclear translocation in HuH7 cells, as evidenced by the overlap of Nrf2 (red) and DAPI (blue) staining (Figure 5a), whereas no such translocation was observed in CA-treated HepG2 cells (Figure 5c). Quantification confirmed that CA significantly increased the percentage of Nrf2-positive nuclei in HuH7 cells but had no effect in HepG2 cells (Figure 5b and d).

### Nrf2 pathway mediates CA-induced inhibition of HuH7 cell proliferation

**Figure 6.**
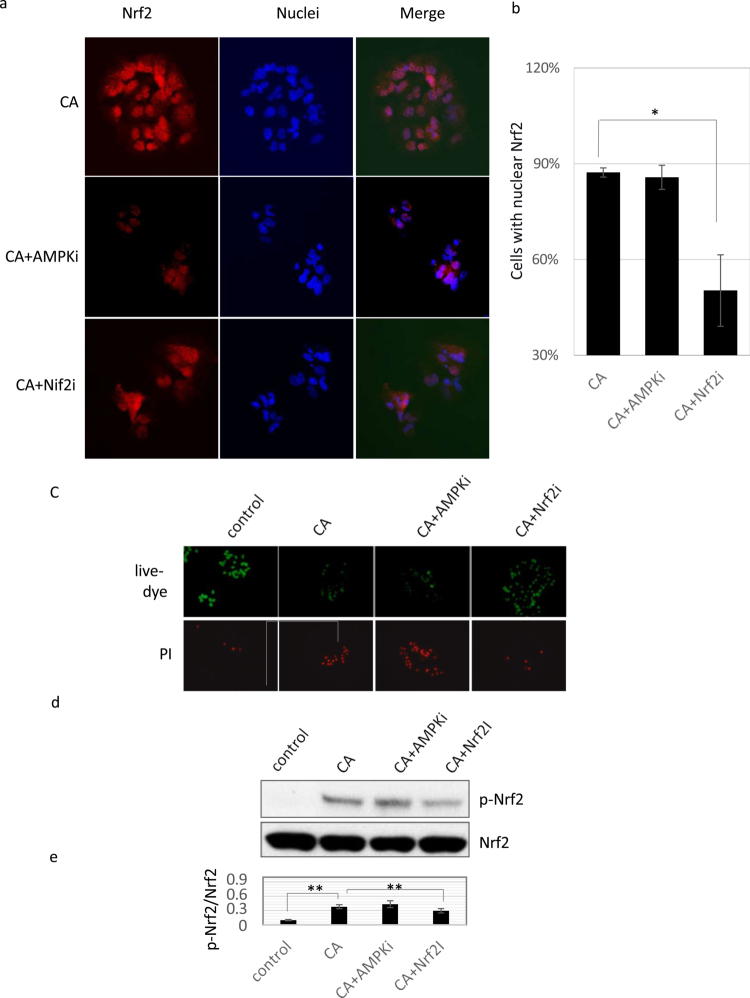
Role of the Nrf2 pathway in CA-induced inhibition of HuH7 cell proliferation. (a) Effect of a specific Nrf2 inhibitor (Nrf2i) compared with a specific AMPK inhibitor (AMPKi) on CA-induced Nrf2 nuclear translocation in HuH7 cells. (b) Quantification of cells with nuclear Nrf2 (%) is shown in (b) (c) Live/dead cell analysis of HuH7 cells treated with CA alone or with either Nrf2i or AMPKi. (d) Western blot analysis showing Nrf2 phosphorylation in response to CA alone or with either Nrf2i or AMPKi. Total protein levels of Nrf2 were used as a control. (e) Ratios of arbitrary values of phosphorylated Nrf2 against total Nrf2 protein levels are shown. Data are expressed as the mean ± SD (*n* = 3). *, *p* < 0.05; **, *p* < 0.001 *vs*. respective control. Tukey's post hoc test was used.

To further confirm the involvement of the Nrf2 pathway in CA-induced inhibition of HuH7 cell proliferation, we used a specific Nrf2 inhibitor (Nrf2i, ML385). CA-induced Nrf2 nuclear translocation (Figure 6a and b), cell death (Figure 6c) and Nrf2 phosphorylation were all significantly inhibited by the Nrf2i (Figure 6d and e). In contrast, pre‑treatment with the AMPK inhibitor compound C (AMPKi) did not affect CA‑induced Nrf2 nuclear translocation (Figure 6a and b), cell death (Figure 6c), or Nrf2 phosphorylation (Figure 6d and e).

## Discussion

Our findings show that Pg and its LPS significantly enhance the proliferation of gastrointestinal cancer cells, including both pancreatic and liver cancer cells. Additionally, our data suggest that the effects of Pg are associated with the upregulation of TLR4 and downstream molecules such as ERK, Akt and NF-κB. These findings confirm previous studies that have identified Pg and Pg-derived LPS as promoters of cancer cell growth in various cell types, through the induction of inflammatory responses and activation of signaling pathways associated with tumorigenesis [21,22]. Moreover, the use of purified Pg-LPS (Figure 3) demonstrates that the pro-proliferative effect is mediated by bacterial components rather than requiring active infection.

Human pancreatic carcinoma cells (RC1973) were included as an additional gastrointestinal cancer cell line for two specific purposes. First, to assess whether the pro-proliferative effect of Pg and its LPS is specific to liver cancer cells or represents a broader phenomenon across gastrointestinal malignancies. Second, to serve as a positive control for method development and validation of Pg infection conditions, based on previous reports that Pg can affect pancreatic cancer cells [23,24]. The inclusion of this second gastrointestinal cancer cell line thus enhances both the credibility and the generalizability of our Pg/LPS findings.

A key and nuanced finding of this study is that the action of CA on liver cancer cells is both cell-type-specific and concentration-dependent. CA inhibited proliferation in HuH7 cells at higher concentrations (≥2 µM) but did not show such an effect in HepG2 cells. Intriguingly, at lower concentrations (≤1 µM), CA exhibited a stimulatory effect on proliferation in both cell lines. This biphasic response—promoting growth at low doses while inhibiting it at higher doses in susceptible cells—has been observed for other natural compounds [25], and it underscores the critical importance of dose optimization in therapeutic applications [26]. The molecular switch governing this transition from pro-survival to anti-proliferative effects warrants deeper investigation.

Our experiments show that CA's effect on HepG2 cells is cell-type-determined rather than bacteria-driven. Accordingly, we focus our discussion on CA's mechanism via Nrf2, not on Pg/LPS signaling. However, the relationship between CA's responses in HuH7 cells and Pg/LPS signaling –particularly whether CA directly modulates Pg/LPS-induced activation of the TLR4/ERK/Akt/NF-κB pathways—warrants further investigation to fully elucidate CA's function in regulating Pg and its signaling. Two negative findings are particularly noteworthy. First, CA did not inhibit LPS-induced proliferation in HepG2 cells (Figure 3). Second, CA had no anti-proliferative effect on HepG2 cells across all concentrations tested (Figure 4b). Rather than being trivial, these observations were pivotal; they refuted our initial expectation that CA might directly counteract Pg/LPS signaling and instead prompted us to explore cell-type-specific mechanisms, ultimately leading to the discovery of Nrf2-dependent inhibition in HuH7 cells.

Our findings indicate that CA induces Nrf2 activation in HuH7 cells but not in HepG2 cells, suggesting that Nrf2 activation plays a critical role in the anti-proliferative effects observed in HuH7 cells at high CA concentrations. Nrf2 is a master regulator of antioxidant responses and has a complex, context-dependent role in cancer, being implicated in both cell survival and cell death under different conditions [27,28]. The inhibition of CA's effects by a specific Nrf2 inhibitor reinforces the idea that the Nrf2 pathway is essential for the observed growth inhibition in HuH7 cells [29]. In contrast, the lack of Nrf2 response in HepG2 cells may indicate a different regulatory mechanism, such as constitutive activation of alternative pathways (e.g. PI3K/Akt or Wnt/β-catenin) that crosstalk with or suppress the KEAP1-Nrf2 axis, rendering these cells resistant to CA-mediated growth modulation [30].

The anti-proliferative effect of CA in HuH7 cells, measured by reduced metabolic activity (Figure 4a, b and c), is accompanied by induction of cell death, as shown by Live/Dead staining (Figure 6c). This suggests that CA may act through both cytostatic and cytotoxic mechanisms in responsive cells. In addition, the lack of effect of AMPK inhibition serves as a key specificity control, strengthening the conclusion that CA acts primarily through Nrf2 in HuH7 cells. While CA has been reported to inhibit liver cell proliferation through AMPK [31], our negative result confirms that the anti-proliferative effects of CA in HuH7 cells are specifically mediated by Nrf2, rather than by AMPK signaling. Together, these findings emphasize the necessity for personalized approaches that consider both the intrinsic molecular landscape of the tumor and precise dosing. The molecular basis for the lack of Nrf2 activation in HepG2 cells remains to be elucidated and warrants further investigation.

While CA can directly influence intrinsic cancer cell proliferation pathways like Nrf2, it may not effectively block the initial pro-inflammatory trigger mediated by external pathogen-associated molecular patterns like Pg-LPS. This observation underscores the distinct layers of intervention—targeting the microbial trigger versus modulating the host cell response—and suggests that combination therapies addressing both aspects might be necessary for optimal efficacy in infection-associated cancers.

The present study is limited to in vitro experiments; future in vivo studies using xenograft or orthotopic mouse models are needed to validate the pro-proliferative effect of Pg and the cell-type-specific action of CA in a more complex tumor microenvironment. Although CA inhibited HuH7 proliferation at higher concentrations, lower concentrations (0.1–1µM, Figure 4c and d) promoted proliferation in both cells. These observations indicate that the mechanisms of CA warrant more comprehensive investigation.

In conclusion, our findings illuminate the complex interactions between the oral pathogen Pg, its LPS and the natural compound CA in modulating liver cancer cell proliferation. We demonstrate that CA's effect is both cell-type-specific and concentration-dependent, with its inhibitory action contingent upon successful Nrf2 pathway activation. These insights contribute to a more nuanced understanding of the oral-systemic link in cancer and highlight the importance of considering cellular context and precise dosing in the application of natural compounds for cancer prevention or adjunctive therapy. Future studies should focus on elucidating the precise molecular determinants of Nrf2 responsiveness to CA across different liver cancer subtypes and validating these interactions in more complex in vivo models of Pg-associated hepatocarcinogenesis.
